# Direct electron beam patterning of electro-optically active PEDOT:PSS

**DOI:** 10.1515/nanoph-2023-0640

**Published:** 2024-01-04

**Authors:** Siddharth Doshi, Dominik Ludescher, Julian Karst, Moritz Floess, Johan Carlström, Bohan Li, Nofar Mintz Hemed, Yi-Shiou Duh, Nicholas A. Melosh, Mario Hentschel, Mark Brongersma, Harald Giessen

**Affiliations:** Department of Materials Science and Engineering, Stanford University, Stanford, CA 94305, USA; 4th Physics Institute and Research Center SCoPE, University of Stuttgart, Pfaffenwaldring 57, 70569 Stuttgart, Germany; Geballe Laboratory for Advanced Materials, Stanford University, 476 Lomita Mall, Stanford, CA 94305, USA

**Keywords:** direct electron beam lithography, metallic polymer, electrically switchable diffraction grating, PEDOT:PSS

## Abstract

The optical and electronic tunability of the conductive polymer poly(3,4-ethylenedioxythiophene):poly(styrene sulfonate) (PEDOT:PSS) has enabled emerging applications as diverse as bioelectronics, flexible electronics, and micro- and nano-photonics. High-resolution spatial patterning of PEDOT:PSS opens up opportunities for novel active devices in a range of fields. However, typical lithographic processes require tedious indirect patterning and dry etch processes, while solution-processing methods such as ink-jet printing have limited spatial resolution. Here, we report a method for direct write nano-patterning of commercially available PEDOT:PSS through electron-beam induced solubility modulation. The written structures are water stable and maintain the conductivity as well as electrochemical and optical properties of PEDOT:PSS, highlighting the broad utility of our method. We demonstrate the potential of our strategy by preparing prototypical nano-wire structures with feature sizes down to 250 nm, an order of magnitude finer than previously reported direct write methods, opening the possibility of writing chip-scale microelectronic and optical devices. We finally use the high-resolution writing capabilities to fabricate electrically-switchable optical diffraction gratings. We show active switching in this archetypal system with >95 % contrast at CMOS-compatible voltages of +2 V and −3 V, offering a route towards highly-miniaturized dynamic optoelectronic devices.

## 1 Introduction

The conductive polymer PEDOT:PSS has attracted broad interest as a promising materials platform for future body-worn technologies. Its conductivity, mechanical compliance, and active optoelectronic tunability have enabled diverse applications ranging from wearable displays [1–3], bioelectronic devices [4–8], to future augmented/virtual reality (AR/VR) systems [9, 10]. The optical and electronic properties of conducting polymers such as PEDOT:PSS can be electrically modulated by modifying the oxidation state of their conjugated polymer backbones [11–15]. This is straightforwardly achieved through electrochemical intercalation of ions from an electrolyte solution. Miniaturization, enabled by high-resolution patterning processes, will be instrumental in realizing the next generation of novel conductive polymer-based devices. The push for higher spatial resolution is especially important for emerging applications in micro- and nanophotonics. The significant tunability of PEDOT:PSS at near-infrared (NIR) and infrared (IR) wavelengths, where it can be switched between metallic and dielectric states, enables dynamic wavefront control [9, 10, 16–19]. Such devices require active sub-units with wavelength or sub-wavelength scale dimensions (<2 µm).

There are currently no straightforward methods to pattern PEDOT:PSS films at this scale. Conventional photolithography is incompatible with fabrication of electro-optically active polymers due to their lack of chemical orthogonality with photoresists and harsh processing conditions [20, 21]. Alternate lithographic processes require tedious and multi-step indirect patterning and dry etching processes [9, 22, 23]. Solution-processed methods such as inkjet printing and screen printing typically have limited spatial resolution (>100 µm) [24–27]. Direct optical patterning of PEDOT:PSS microstructures, with minimum line widths of 6 µm [28] and 2 µm [29], has recently been achieved through the use of formulations that include light-sensitive additives. However, these promising advances still do not meet the requirements of active micro and nano-photonic devices. Furthermore, it is unclear how their optical properties are affected by inclusion of additives such as UV cross-linkable monomers.

**Figure 1:**
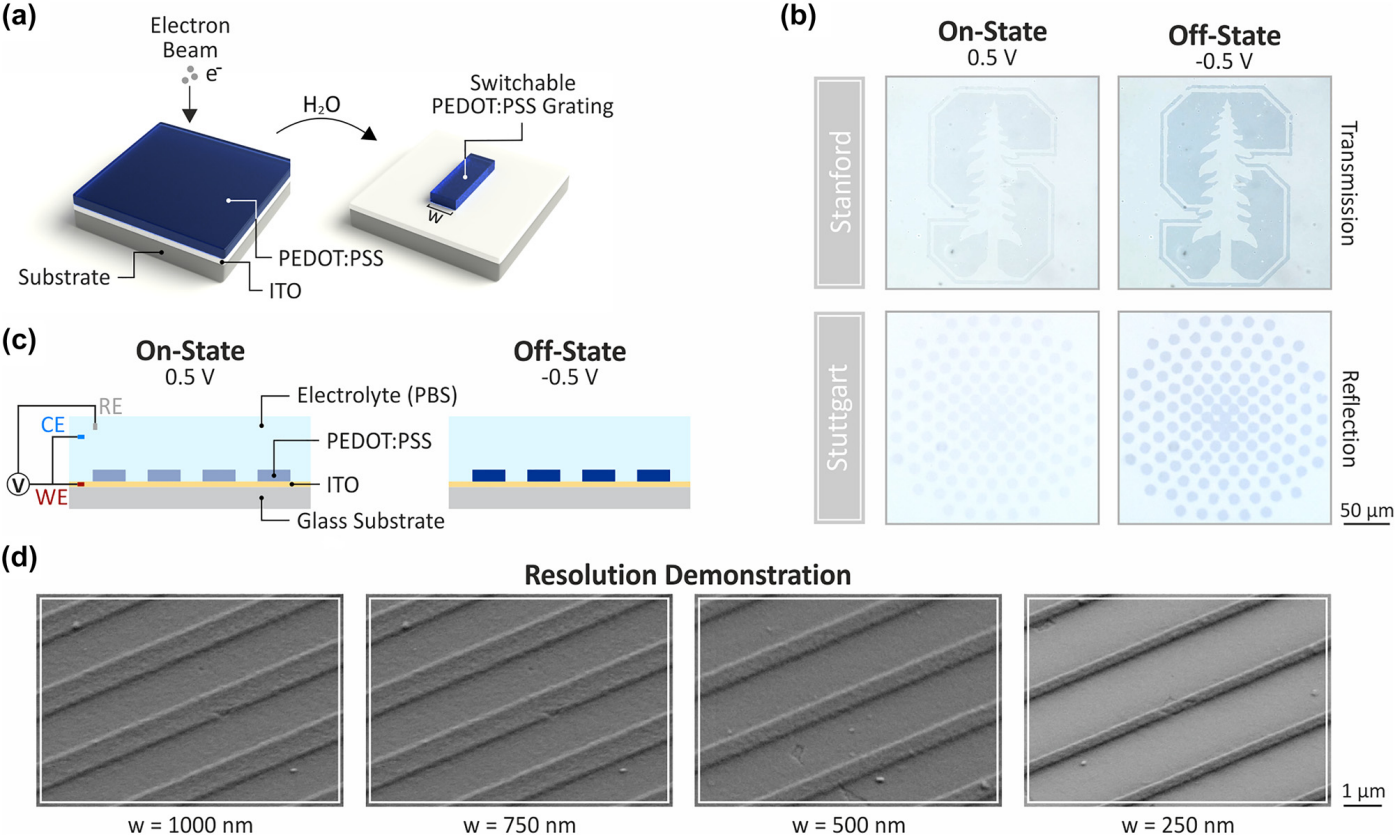
High resolution, direct patterning of PEDOT:PSS via electron beam induced solubility modulation. (a) Schematic illustration of the fabrication workflow for direct electron beam lithography (EBL). The metallic polymer PEDOT:PSS is spin-coated onto a 20 nm indium-tin-oxide (ITO) coated glass substrate. No additional heat- or post-treatment is required. The metallic polymer is directly patterned via electron beam lithography. After the sample has been developed for 90 s in H_2_O to remove the unexposed material, an electrically switchable metallic polymer structure is obtained. (b) Schematic of the electrochemical setup and the switching behavior. Dark blue indicates the insulating state. (c) Switching of visible electrochromism between the on- and off-state is demonstrated with optical micrographs of a patterned Stanford logo measured in transmission (upper) and a patterned Stuttgart logo measured in reflection (lower) upon switching of the applied potential between 0.5 V and −0.5 V in a three electrode electro-chemical set-up in the aqueous electrolyte, phosphate buffered saline (PBS). The voltage is applied to the working electrode (WE), an ITO coated glass substrate on which the structures are fabricated, relative to an Ag/AgCl reference electrode (RE), with a Pt wire serving as a counter-electrode (CE). The voltage change leads to a color variation visible in the two logos. (d) Scanning electron microscope (SEM) images to demonstrate the achievable resolution with metallic polymer gratings produced via our direct fabrication scheme. The periodicity for all gratings is 3 µm while the width *w* of the wires is reduced from 1 µm to 250 nm. The film thickness of the PEDOT:PSS layer is 90 nm for all measurements.

To address these limitations, we report a process for high-resolution, direct electron beam patterning of PEDOT:PSS. Our process involves selectively exposing a film of commercially available PEDOT:PSS with a pattern using an electron beam lithography (EBL) tool, followed by a simple development in water (Figure 1A). In its pristine state, PEDOT:PSS is soluble in water. Exposure to electron beam radiation reduces its solubility [30]. Therefore, water-soluble unexposed regions are washed away during development, leaving only the EBL-patterned regions. The simplicity of our approach, where we use PEDOT:PSS as a negative electron beam resist, could greatly streamline the fabrication of active polymer devices.

We take advantage of the high resolution of EBL to write nano-wire features with a line width of 250 nm, almost an order of magnitude finer than prior direct write approaches. We find that our EBL process maintains the conductivity of PEDOT:PSS relative to unexposed polymer and that our treatment is compatible with inclusion of the common conductivity enhancing additive, ethylene glycol [31–34]. This allows us to write PEDOT:PSS with conductivities of up to 460 S/cm. Furthermore, the EBL written structures are stable in aqueous electrolytes, where their electrochemical impedance is similar to a chemically crosslinked PEDOT:PSS formulation [5, 35] widely used in bioelectronics.

Finally, we illustrate the potential of our process to enable novel technologies by demonstrating micro-fabricated active photonic devices. We find that the optical properties of EBL exposed PEDOT:PSS are similar to unexposed polymer, exhibiting metal-like reflectivity in the IR [9, 36]. We use our high-resolution writing capabilities to fabricate electrically switchable infrared diffraction gratings. Our devices can be switched “ON” and “OFF” with >95 % switching contrast by application of CMOS-compatible voltages in a solid-state gel electrolyte, highlighting their applicability for the next generation of low-power, dynamic optoelectronic systems.

## 2 Results and discussion

### 2.1 Direct writing of functional PEDOT:PSS structures with sub-μm resolution

Our process directly patterns PEDOT:PSS through electron-beam-induced solubility modification. In its pristine state, PEDOT:PSS is soluble in water. Prior studies have reported that electron beam radiation reduces its solubility. This change has been attributed to radiation-induced crosslinking caused by electron-beam exposure [30]. We hypothesize that we can leverage well-established EBL tools to differentially modulate the solubility of PEDOT:PSS at a high spatial resolution. We find that upon exposure of a spin-coated film to a sufficiently high electron-beam dose, EBL-patterned regions are insoluble, while unexposed polymer remains soluble and is washed away upon a brief immersion in water. In this process, PEDOT:PSS serves as a negative electron beam resist with a facile, single-step development in water (Figure 1A). In comparison to current tedious and multi-step lithographic processes for patterning of PEDOT:PSS, our direct-write approach greatly simplifies process flows while avoiding exposure to harsh solvents and process conditions [9, 20–23]. We illustrate our ability to deterministically write target patterns by re-creating the Stanford and Stuttgart logos (Figure 1B) from PEDOT:PSS films with a thickness of 90 nm. We use this film thickness for all subsequent measurements.

We confirm the functionality of our written structures by demonstrating electrochromic switching of our patterned logos. The unique properties of PEDOT:PSS arise from its ability to couple ionic and electronic charges. Electrochemical intercalation of positive ions from electrolyte solution into the bulk of the material results in depletion of hole charges along the conjugated polymer backbone, modifying its band-structure and associated optical transitions. This results in a change in the optical and electronic properties of PEDOT:PSS, enabling a range of applications including electrochromic displays and organic electrochemical transistors (OECTs). To demonstrate that the written structures retain this functionality, we electrochemically modulate the optical properties of our logo structures using a three-electrode electrochemical set-up in the aqueous electrolyte, phosphate buffered saline (PBS). As we switch the external voltage from +0.5 V to −0.5 V, the absorbance of the material increases at visible wavelengths, resulting in a change in color (Figure 1B), transmittance, and reflectance (Figure S4) as expected for PEDOT:PSS.

A key advantage of EBL is its ability to pattern high resolution features. We find that our process allows for writing of prototypical nano-wire structures with a line width as fine as 250 nm. At finer line widths, adhesion to the substrate is reduced. Consequently, a resolution limit of 250 nm is achieved. This resolution is almost an order of magnitude finer than previously reported direct write approaches with minimum line widths of 6 µm [28] and 2 µm [29] that are based on photo-patterning of PEDOT:PSS formulations with light-sensitive additives. This could open opportunities for a range of novel chip-scale optical and electronic devices. To evaluate the potential of this method, we first characterize the optical and electronic properties of EBL-patterned PEDOT:PSS and compare it to the pristine unexposed polymer.

### 2.2 Electrical and electrochemical properties of EBL written PEDOT:PSS

**Figure 2:**
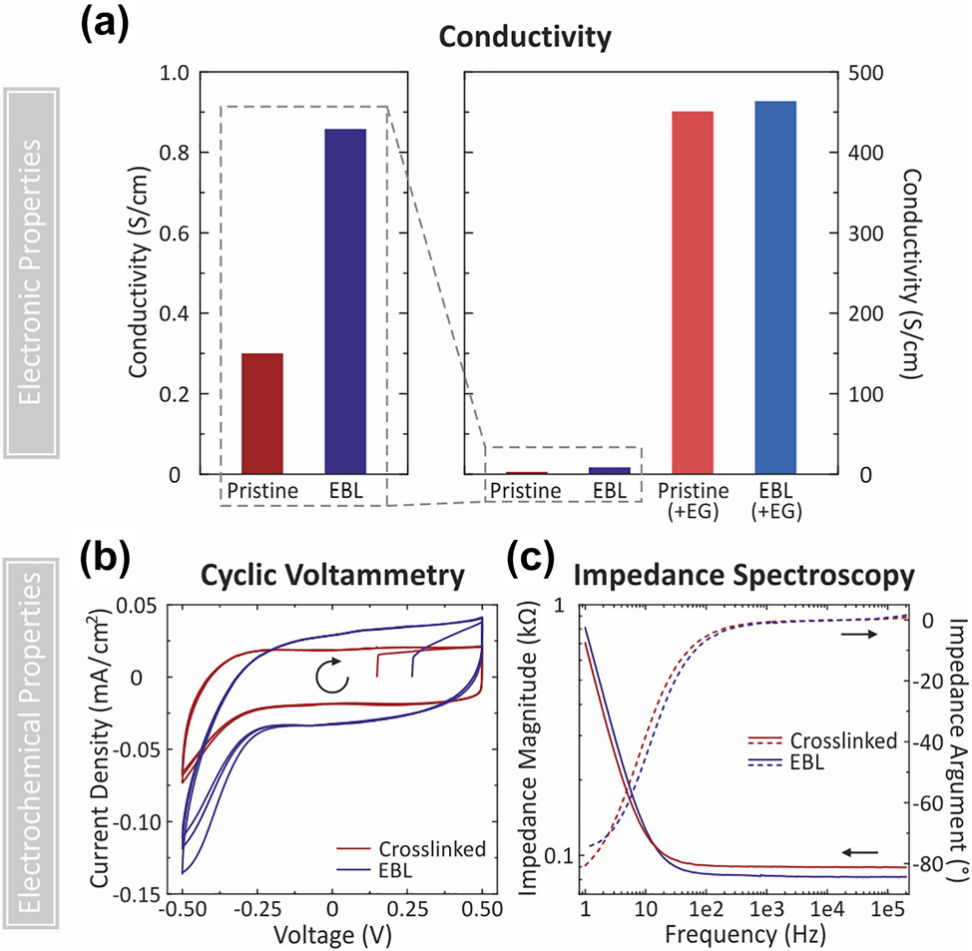
Comparison of conductivity and electrochemical properties of pristine and chemically crosslinked (red) with EBL exposed (blue) PEDOT:PSS. (a) Conductivity of pristine and EBL exposed PEDOT:PSS samples as determined by a 4-point probe measurement without any additives (left) and with inclusion of the additive, ethylene glycol, common used to enhance conductivity (right). (b and c) Electrochemical properties of EBL exposed PEDOT:PSS in phosphate buffered saline solution compared to PEDOT:PSS chemically crosslinked with the standard chemical cross-linker, (3-glycidyloxypropyl)trimethoxysilane, studied through (b) cyclic voltammetry and (c) electrochemical impedance spectroscopy, represented through a Bode plot. Measurements were conducted in a custom-built electrochemical cell. Gold (70 nm, 5 nm Ti adhesion layer) coated Si was used as a substrate and working electrode, an Ag/AgCl pellet electrode was used as a reference electrode and a platinum wire was used as a counter electrode.

PEDOT:PSS has emerged as an ideal electrode material for transparent electronic devices and bioelectronic interfaces. This is driven by its high conductivity and low electrochemical impedance. We find using four-point-probe measurements that our EBL process maintains the conductivity of PEDOT:PSS relative to unexposed polymer (Figure 2A). The conductivity of EBL exposed polymer (0.85 S/cm) is more than 2× that of pristine PEDOT:PSS (measured as 0.3 S/cm, consistent with prior work [31–34]). Future work could investigate the mechanism of this conductivity enhancement. For many applications in electronics and bioelectronics, PEDOT:PSS formulations incorporating conductivity-enhancing additives are widely utilized to improve the conductivity by a factor of over 1000 [31–34]. To evaluate whether conductivity improvements from additive-enhanced formulations are maintained upon EBL patterning, we pattern films that include the common conductivity enhancing additive, ethylene glycol (EG). We are able to direct-write highly conductive PEDOT:PSS structures with conductivities of up to 463 S/cm, similar to un-exposed EG-enhanced films (450 S/cm). More broadly, there is a vast body of literature reporting on chemical additives that modify the properties of PEDOT:PSS [37]. Investigating how various additives and formulations of PEDOT:PSS are affected by EBL-patterning could allow for the direct writing of functional polymer nanostructures with a broad range of achievable electrical, optical, and mechanical properties.

When used as an electrode interface for biological cells such as neurons, PEDOT:PSS structures must be stable in aqueous solution. As PEDOT:PSS is soluble in water, aqueous stability is typically achieved through the use of chemical cross-linkers such as (3-glycidyloxypropyl)trimethoxysilane (GOPS), introducing additional challenges for patterning of PEDOT:PSS [5, 35]. In contrast, EBL-patterned structures are water-stable without any further treatment, as demonstrated in Figure 1D. We evaluate the electrochemical behavior of EBL-patterned PEDOT:PSS by carrying out cyclic voltammetry (CV) and electrochemical impedance spectroscopy (EIS) of our structures in PBS. Experiments are conducted with large area patterns using a custom built electrochemical cone cell (Figure S3), and results are compared with standard GOPS-crosslinked PEDOT:PSS films. We find that the CV curves (Figure 2B) for GOPS-crosslinked films and EBL films are similar, with predominantly capacitive behavior and a tail at negative voltages due to oxygen reduction. The impedance, as shown in the Bode plot in Figure 2C, is similar for both films, indicating its suitability for use as a low-impedance electrode material.

By greatly enhancing process flexibility for the high-resolution patterning of conductive, low-impedance PEDOT:PSS films, we could enable a new generation of miniaturized electronic and bioelectronic devices. Examining the switching of electronic properties could enable future applications in active OECT’s, for use in sensors and computing devices. Additionally, establishing the compatibility of our process with flexible substrates (e.g. PET) could enable application in future flexible wearable devices.

### 2.3 Optical properties

**Figure 3:**
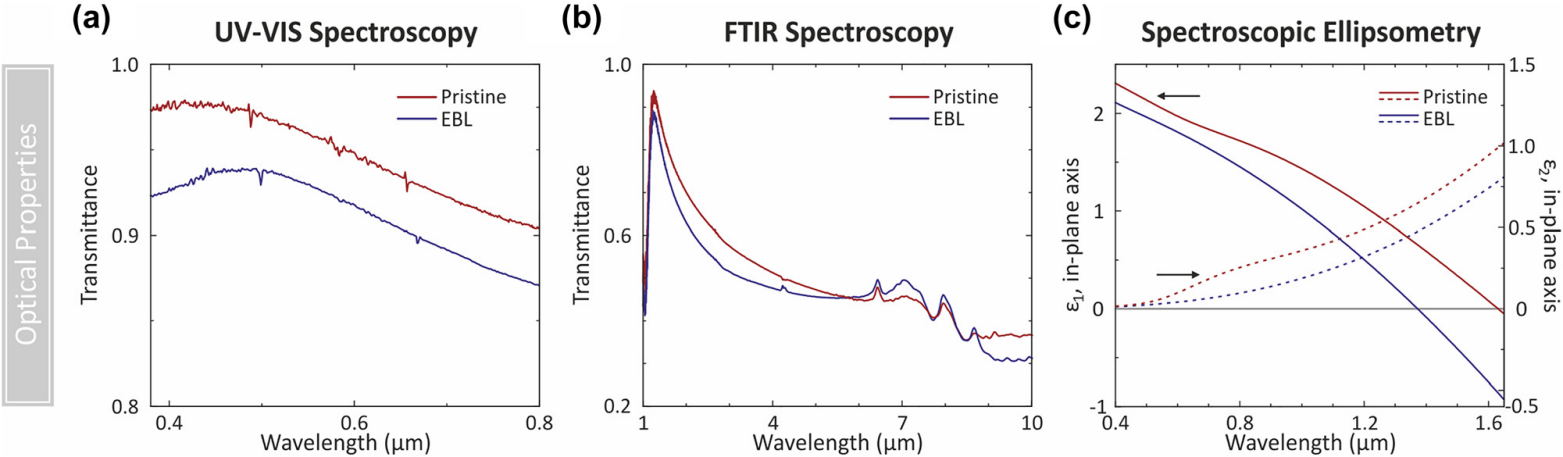
Comparison of optical properties of pristine (red) and EBL exposed (blue) PEDOT:PSS. (a and b) Transmission spectra measured with UV–vis spectroscopy and Fourier-transform infrared (FTIR) spectroscopy. The pristine and the EBL exposed film is about 90 nm in thickness. In (a) the transmittance was measured between 380 nm and 800 nm. The reference signal was measured through the bare glass substrate. In (b) the transmittance was measured between 1 µm and 10 µm. For this measurement the PEDOT:PSS film was spin-coated on a double-side polished silicon wafer and the reference signal was measured through the bare silicon substrate. (c) Resulting optical constants derived from variable angle spectroscopic ellipsometry measurements. Here, the real part *ε*_1_ (solid line) and imaginary part *ε*_2_ (dotted line) of the dielectric function *ε* = *ε*_1_ + i*ε*_2_ are presented for the metallic polymer PEDOT:PSS in the in-plane sample axis in the pristine and EBL exposed state. In the ellipsometry measurements, four angles of incidence were used for the pristine material and two angles were used for the EBL exposed material. To obtain a good agreement between the measurement and the model, a combination of spectroscopic ellipsometry and transmission data was used. Both models were generated between 400 nm and 1650 nm and are based on an anisotropic generalized oscillator approach. For the pristine PEDOT:PSS, a mean square error (MSE) of 2.2, and for the EBL exposed material an MSE value of 8.8 was obtained. For the pristine state, a metallic polymer with *ε*_1_ < 0 is obtained for *λ* > 1.6 µm. For the EBL-exposed material, metallic properties with *ε*_1_ < 0 are obtained for *λ* > 1.35 µm.

We find that the optical properties of EBL exposed structures are similar to pristine PEDOT:PSS. At visible wavelengths, PEDOT:PSS is widely used as a transparent conductor. We find that overall, EBL-written PEDOT:PSS maintains this transparency, although the transmittance of the structures is reduced by between 3 and 5 % at different wavelengths in the visible spectrum between 380 and 800 nm (Figure 3A). The film thickness in both cases is ∼90 nm, indicating that this reduced transmittance stems from higher material absorption. Future work could further investigate the processes that lead to this change in absorptivity, which could range from electron-beam induced changes in oxidation state to changes in the fraction of water-soluble PSS present in the film after development. At infrared wavelengths, where PEDOT behaves as a lossy metallic film [9, 36] the Fourier-transform infrared (FTIR) spectra for EBL and pristine PEDOT:PSS are similar (Figure 3B).

We perform variable angle spectroscopic ellipsometry (VASE) to compare the optical constants of pristine and EBL-exposed PEDOT:PSS between 400 and 1650 nm (Figure 3C). We developed a uniaxial anisotropic model to consider the materials optical response in both in-plane and out-of-plane directions (Figure S1), and further constrained our fitting using transmission measurements. A Drude oscillator and a Gaussian oscillator were used to model the optical response of the pristine polymer, while only a Drude oscillator was required to achieve good agreement with the measured data for the EBL-exposed material.

A material exhibits metallic optical properties at frequencies below its plasma frequency when the real part of its dielectric constant *ε*_1_(*ω*) is negative. Pristine PEDOT:PSS exhibits metallic behavior (*ε*_1_ < 0) at *λ* > 1.6 µm, as compared to *λ* > 1.35 µm for EBL-exposed PEDOT:PSS. The blue shift of the plasma frequency, (where *ε*_1_ = 0), for EBL-exposed PEDOT:PSS indicates that either the carrier density or mobility have increased. This is consistent with prior conductivity results, which indicate that the DC conductivity has increased for EBL-exposed PEDOT:PSS. Further studies, including Hall-effect measurements, could help to distinguish between changes in carrier density and mobility and provide insight into the fundamental properties of EBL-exposed PEDOT:PSS.

### 2.4 Electrically switchable infrared diffraction gratings

The tunability of the optical properties of PEDOT:PSS at near-infrared (NIR) and infrared (IR) wavelengths, where it can be electrochemically switched between metallic and dielectric states, has attracted significant interest in micro- and nano-optics. Actively modulating the properties of micro- and nano-structured optical elements allows for dynamic control of optical wavefronts, enabling a host of applications including tunable lenses and holographic displays [9, 10, 16–19, 38–43]. These devices require active sub-units with wavelength or sub-wavelength scale dimensions (<2 µm). Electrically switchable PEDOT:PSS nano-antennas as fine as 180 nm had been fabricated using conventional electron-beam lithographic processes [9, 10, 16, 19]. However, the tedious multi-step process leaves a SiO_2_ hard mask on top of the fabricated structures, potentially modifying the optical properties of the structures and limiting access to electrolyte, rendering novel fabrication approaches highly desirable. Other previously discussed direct write methods are unable to achieve the required feature sizes for such applications [28, 29]. The push for novel methods to achieve sub-wavelength spatial resolution is therefore especially important for emerging applications in micro- and nanophotonics. Our fabrication process, which can achieve 250 nm linewidths while maintaining the metallic properties of PEDOT:PSS in its unmodulated dry state, would accelerate the development of conductive polymer based active micro- and nano-optics. This could include hybrid systems that couple dielectric and plasmonic metasurfaces with switchable conductive polymers [44], or entirely polymer-based active optoelectronic devices.

**Figure 4:**
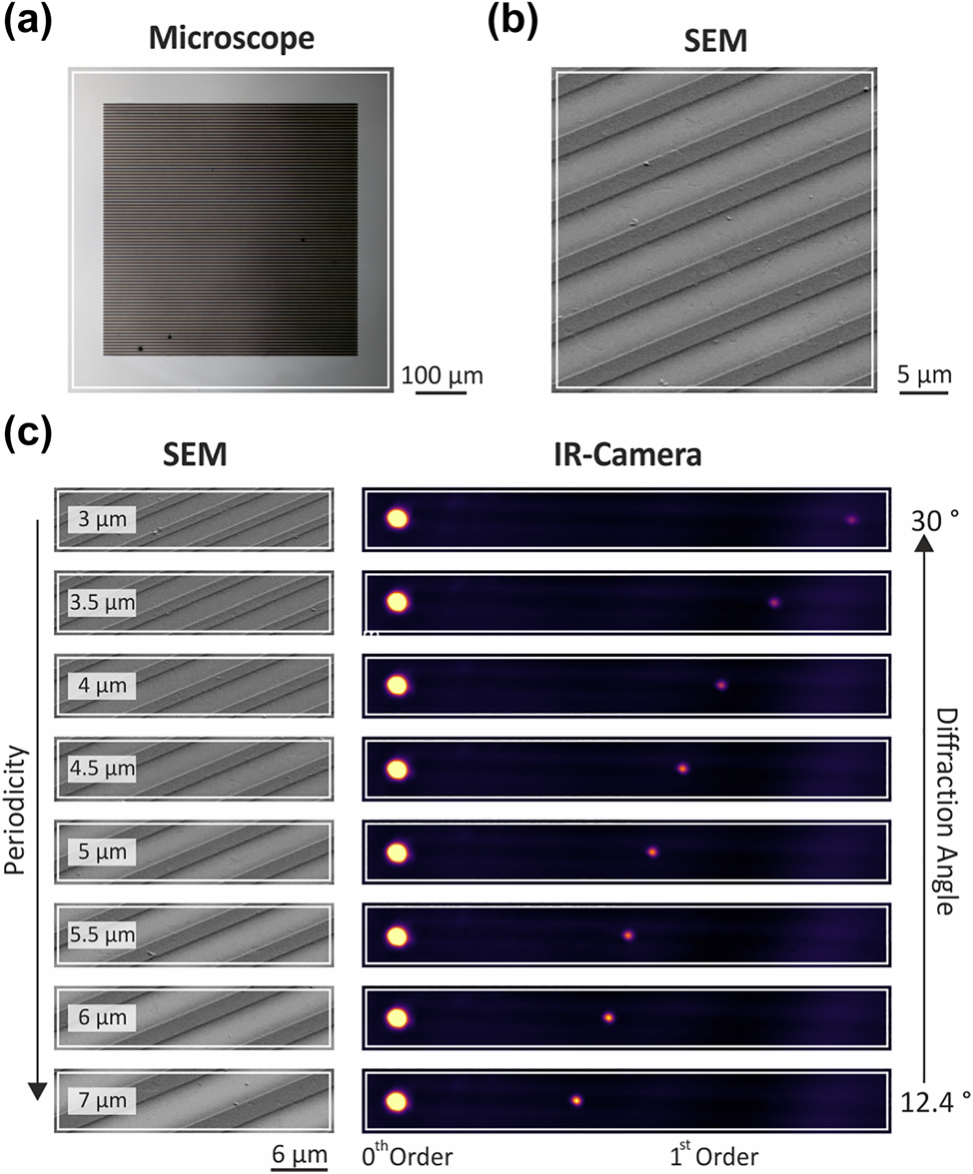
Conducting polymer PEDOT:PSS diffraction grating and diffraction angle variation by changing the periodicity. Presentation of the fabricated diffraction gratings via (a) a microscope image and (b) an SEM image of a section of the structure. The length and width of the diffraction grating arrays are 500 µm. In (b) the periodicity is 6 µm and the width of the metallic polymer wires is 2 µm. (c) Variation of the diffraction angle between 30° and 12.4° by changing the periodicity and width of the diffraction grating while keeping the aspect ratio constant at 1/3. The periodicity is increased from 3 µm to 7 µm and the width of the wires is adjusted accordingly. The zeroth- and first-order are clearly visible in the IR camera images and by increasing the periodicity the diffraction angle is decreased. To prevent saturation of the IR camera the transmitted beam is attenuated. The measurement is performed at a wavelength of 1.5 µm.

We illustrate the potential for our high-resolution direct writing process to enable novel micro-optic components by first writing chip-scale arrays (500 µm × 500 µm) of PEDOT:PSS micro-wires to form infrared diffraction gratings (Figure 4A and B). We investigate the behaviour of our system using an IR camera to capture the diffraction pattern generated by the PEDOT:PSS grating at a wavelength of 1.5 μm. PEDOT:PSS exhibits metallic optical properties at 1.5 µm, allowing our gratings to strongly interact with incident light waves and re-direct them into the first diffracted order, as observed in IR camera images (Figure 4C). We note that the zeroth-order beam is attenuated to prevent saturation of the IR camera. We show that we can vary the diffraction angle by modifying the periodicity while keeping the fill factor constant. Nano-wire arrays with a 3 µm periodicity and 1 µm line-width diffract at an angle of 30°, while arrays with a 7 µm periodicity diffract at an angle of 12.4°.

**Figure 5:**
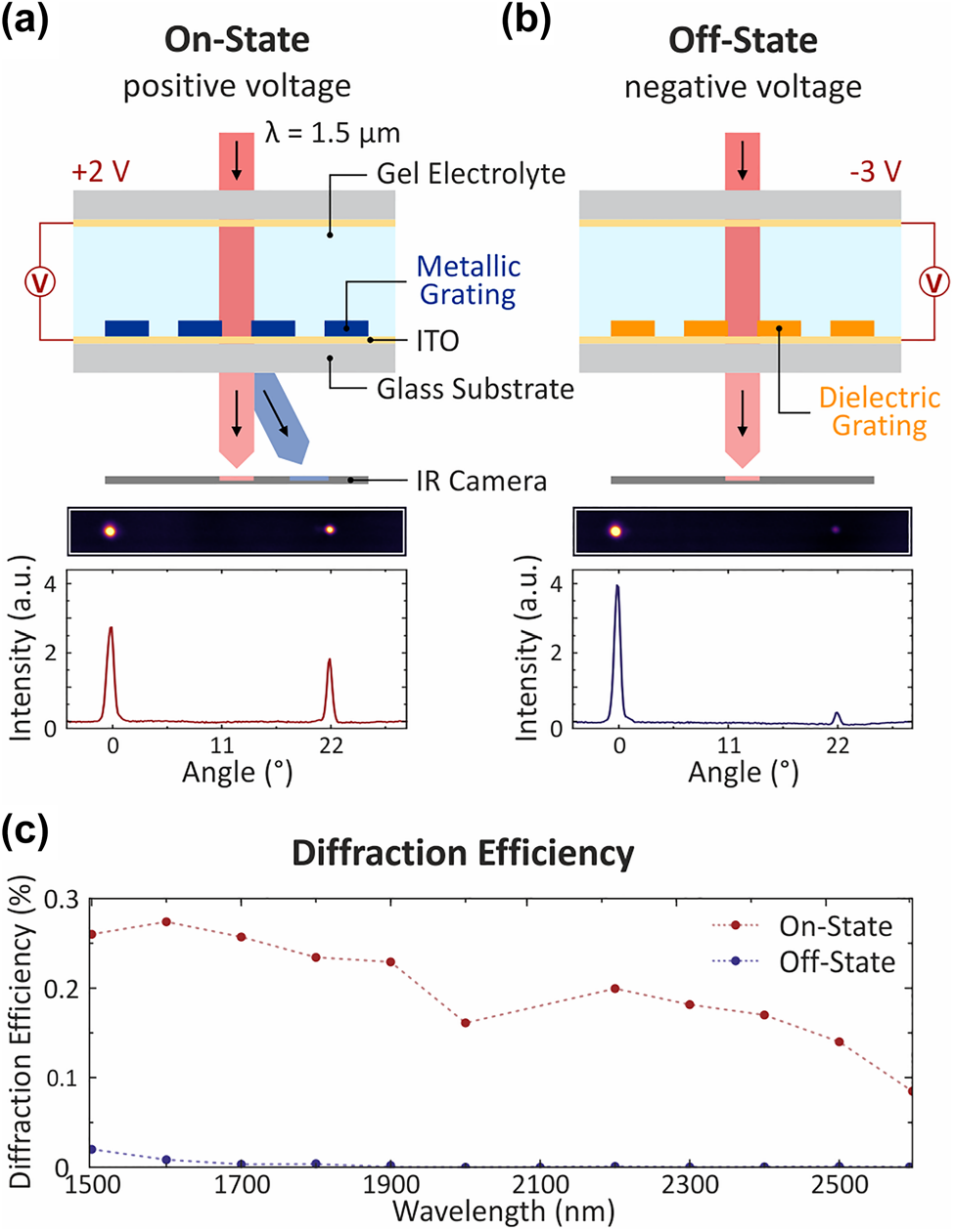
Electrochemical switching of conducting polymer diffraction gratings and analysis of the diffraction efficiency for wavelengths between *λ* = 1.5 μm to 2.6 μm. (a and b) (Top) Schematic of the electrochemical cell based on two ITO-coated glass substrates used for electrical contact encapsulating the fabricated diffraction grating combined with the gel electrolyte. In the on-state (a), a positive voltage of +2 V is applied and an increased charge carrier density in the material leads to metallic properties. In (b) the applied voltage is changed to a negative voltage of −3 V, resulting in a significantly reduced charge carrier density in the material. Consequently, the diffraction grating becomes dielectric and the system is turned off. To capture the diffraction pattern generated by the metallic polymer grating at a wavelength of 1.5 μm an IR camera is used. (Bottom) To demonstrate the switching between the on- and the off-state, selected IR camera images and the according intensity profiles are utilized. In the on-state (a) the metallic grating leads to a visible transmitted (red) and diffracted (blue) beam. The diffraction angle for the first-order diffracted beam for a grating with a periodicity of 4 μm and a linewidth of 1.33 μm is 22°. Changing to a negative voltage, the system is turned off (b) and the diffracted beam is only barely visible on the IR camera image. To avoid saturation of the IR camera the transmitted beam is attenuated. (c) Additionally, the diffraction efficiency in the on- (red) and off-state (blue) is analyzed between 1.5 μm and 2.6 μm. The efficiency (calculated as the intensity of the first-order divided by the zeroth-order) is measured for a diffraction grating with a periodicity of 7 μm. For this measurement, no attenuation of the transmitted beam is used anymore.

We finally demonstrate active switching of these micro-optic devices through electrochemically induced carrier density modulation. By applying a negative electrochemical potential, we deplete hole charge carriers in the conjugated polymer backbone as described previously. This reduction in charge carrier density modulates the optical properties of PEDOT:PSS in the infrared, switching it from a metallic state, where the diffraction grating is “ON”, to a dielectric state where the diffraction grating is “OFF” (Figure 5A and B). Furthermore, to illustrate the potential for achieving integrated solid-state devices, we use an ion-gel as the electrolyte. We use a two-electrode electrochemical cell with ITO-coated glass substrates, separated by an ion-gel electrolyte [19], as the working- and counter-electrodes. We measure the resultant diffraction pattern with an IR camera and calculate the diffraction efficiency as the intensity of the first-order divided by the zeroth-order beam. We find that our devices can be almost completely switched from a metallic “ON” state at +2 V, to a dielectric “OFF” state at −3 V, with over >95 % switching contrast at 1.5 µm (Figure 5A and B). The absolute diffraction efficiency is below 1 %. This behavior is maintained throughout the wavelength range of 1.5–3 µm, with slightly lower contrast and efficiencies at longer wavelengths (Figure 3C). While many metasurface tuning approaches based on materials such as liquid crystals, transparent conductive oxides, and phase change materials have been reported, such devices typically operate in voltage ranges between 10 and 100 V, which keeps emerging applications such as in wearables and AR/VR out of reach. In comparison, our devices operate at CMOS-compatible voltages in a solid-state gel based device, highlighting the applicability of our fabrication method towards enabling the next generation of low-power, dynamic optoelectronic systems.

## 3 Conclusions

We have demonstrated a straightforward, direct-write method to nano-pattern commercially-available PEDOT:PSS with feature sizes down to 250 nm using EBL. Our treatment maintains the optical, electronic, and electrochemical properties of PEDOT:PSS and allows for functional electro-optic switching. We believe that our method, which involves a single EBL-exposure followed by a facile development in water, could greatly simplify process flows for a range of existing optical and electronic devices. Additionally, our ability to write high-resolution features an order of magnitude finer than existing direct write approaches could enable a broad range of novel miniaturized systems. As a prototypical demonstration, we fabricate electrically switchable optical diffraction gratings that can be switched “ON” and “OFF” at CMOS-compatible voltages of +2 V and −3 V in a solid-state device. We thereby illustrate the potential of our method for enabling the next generation of highly miniaturized, dynamic opto-electronic devices.

## 4 Experimental section

### 4.1 Materials

All processes were developed using commercial PEDOT:PSS (Clevios PH1000, 1.3 wt percent) purchased from Ossila. Ethylene glycol, 4-dodecylbenzenesulfonic acid (DBSA), (3-glycidyloxypropyl)trimethoxysilane (GOPS), polyethylene glycol (PEO), lithium perchlorate (LiClO_4_), acetonitrile and phosphate buffered saline (PBS, 1×, pH 7.4) were used as received from Sigma-Aldrich. A formulation of chemically cross-linked PEDOT:PSS was prepared by addition of GOPS (1 vol%) to a PEDOT:PSS solution, followed by ultra-sonication for 1 min. To prepare a highly conductive PEDOT:PSS blend, ethylene glycol (5 vol%) and 1 drop of DBSA were added to a PEDOT:PSS solution, followed by ultra-sonication for 1 min.

### 4.2 Substrate preparation

Before spin-coating, substrates are cleaned in acetone and isopropyl alcohol (IPA), followed by 11 min of UV ozone cleaning. The substrate is hydrophilic after UV-ozone treatment, improving the adhesion of PEDOT:PSS. PEDOT:PSS solution is then spin-coated onto the substrate. Samples are allowed to dry in an ambient environment without post-baking or further chemical treatments.

Actively switchable optical devices operating in the visible (Figure 1) and infrared (Figures 4 and 5) were prepared from PEDOT:PSS films spin-coated onto indium tin oxide (ITO) coated glass substrates. Samples for conductivity (Figure 2A) and optical transmission (Figure 3A) measurements were prepared on glass. Electrochemical characterization (Figure 2B and C) was conducted on PEDOT:PSS films spin-coated on an Au-coated Si substrate, prepared by thermal evaporation of Ti (3 nm) followed by Au (100 nm) onto bare Si. FTIR measurements were carried out on samples prepared on bare double-sided polished Si.

### 4.3 Electron-beam lithography

EBL-exposed structures were prepared using the Raith Voyager electron-beam lithography system. Actively switchable infrared gratings were exposed using an acceleration voltage of 20 kV combined with an aperture of 60 µm. For this fabrication process, an EBL beam current of 1240 pA was measured and a constant nominal area dose of 325 μC/cm^2^ was utilized. At this area dose, the best structure quality was obtained. Lower doses lead to partially developed structures, while higher doses result in fully developed structures but with larger proximity effects that limit the achievable resolution. All other samples were prepared with an acceleration voltage of 50 kV and an aperture of 60 µm. To prepare large area samples (>1 mm) for conductivity and electrochemical property characterization, a beam current of 16 nA was used at a nominal area dose of 500 μC/cm^2^. After the patterned electron-beam exposure, samples were developed in deionized water (DI water) for 90 s followed by drying with a nitrogen gun. We note that there is no upper limit for the development period.

### 4.4 Conductivity measurements

Samples were prepared from PEDOT:PSS formulations spin-coated onto glass substrates. Both unmodified PEDOT:PSS and a conductive PEDOT:PSS formulation (with ethylene-glycol) were spin-coated (2000 RPM) to form ∼90 nm thick films. First, we measure the sheet resistance of the PEDOT:PSS films without EBL-patterning using a four-point probe tool (Ossila). To measure the sheet resistance of exposed PEDOT:PSS, we write large rectangles (6 mm × 2 mm) using electron beam lithography. The sheet resistance of these finite structures is extracted using established empirical size corrections [45].

### 4.5 Electrochemical characterisation

Samples were prepared from PEDOT:PSS formulations spin-coated onto Au-coated Si substrates. Both unmodified PEDOT:PSS (2000 RPM) and a chemically cross-linkable PEDOT:PSS formulation with GOPS (3000 RPM) were prepared. Samples prepared from an unmodified blend were subsequently EBL-patterned into a large area 11 mm × 11 mm square. The chemically cross-linkable PEDOT:PSS was then baked at 120 °C for 20 min to induce chemical cross-linking.

We tested samples in a custom-built Teflon capture cell (Figure S3) with an exposed area of 12 cm^2^. We used PBS as an electrolyte. Measurements were taken with a platinum wire counter electrode and an Ag/AgCl pellet reference electrode. Electrochemical Impedance Spectroscopy (EIS) was carried out from 1 Hz to 1 MHz with a 1 mV variation around the open circuit voltage (OCV) of the material. Cyclic voltammetry was carried out by recording the measured current against applied voltage (vs. Ag/AgCl), cycled at a scan rate of 50 mV/s.

### 4.6 Transmittance measurements

The transmittance measurement for the UV–vis range (380–800 nm) was performed in the J.A. Woollam RC2-UI vertical ellipsometer at normal incidence on the sample. For this measurement, the conducting polymer was spin-coated onto a glass substrate resulting in a 90 nm film thickness. For the EBL treated sample a 7 mm × 7 mm area was prepared using EBL. As reference, an uncoated glass substrate was utilized in both measurements. For the FTIR measurement between 1 µm and 10 µm, the conducting polymer was spin-coated onto a double-side polished silicon wafer. Here, the film thickness was again ∼90 nm. For the FTIR measurement, a 100 µm × 100 µm area was exposed to the electron-beam. As reference, the bare silicon substrate was utilized.

### 4.7 Variable angle spectroscopic ellipsometry

The VASE measurement was performed using a J.A. Woollam RC2-UI vertical ellipsometer. For the pristine PEDOT:PSS, the sample was spin-coated onto a glass substrate and the angle-of-incidence (AOI) was varied from 60° to 80° in steps of 5°. To characterize the EBL-exposed material, PEDOT:PSS was patterned into a 7 mm × 7 mm square. Measurements were carried out at two AOIs of 60° and 65°. A measurement with larger AOIs was not possible as the beam diameter became too large and exceeded the exposed area. In both cases, a uniaxial anisotropic generalized oscillator approach was utilized for the optical model. To model the in-plane optical response, a Drude oscillator and a Gaussian oscillator were used for pristine PEDOT:PSS, while only a Drude oscillator was required for the EBL-exposed material. The out-of-plane optical responses of the material could be modeled using only a pole in the UV for both the pristine and EBL-exposed material. We were able to achieve low mean squared errors (MSE) for both materials indicating reliable results for the modeling. In the pristine state, the lowest possible MSE value was 2.2 related to a film thickness in the model of 77.8 nm compared to an MSE of 8.8 achieved for the EBL exposed material with a film thickness of 102.3 nm. The determined film thicknesses are in acceptable agreement with the expected thickness of ∼90 nm.

### 4.8 Electrically-switchable infrared gratings

PEDOT:PSS nano-wire structures are fabricated on a 20 nm ITO coated glass substrate. The polymer is spin-coated using 2500 RPM for 5 s combined with 3000 RPM for 55 s resulting in a film thickness of about 90 nm. No additional post- or heat-treatment is required. Here, the conducting polymer is directly exposed to the electron-beam using an acceleration voltage of 20 kV with an aperture of 60 µm. The home-built electrochemical cell is created using a second ITO coated glass substrate embedding the gel electrolyte. This electrolyte consists of polyethylene glycol (PEO) combined with lithium perchlorate (LiClO_4_) (20:1 ratio) dissolved in acetonitrile. Here we are using a two-electrode setup in which the working-electrode (ITO layer) is in direct contact with the fabricated PEDOT:PSS wires. The counter-electrode is formed by the second glass substrate. A potentiostat (BioLogic SP-200) is used to adjust the applied voltage between +2 and −3 V.

### 4.9 Visible electro-chromic switching

We use a home-built electrochemical flow cell to allow for electrochemical switching of the polymer under a microscope objective with a three-electrode setup. Flow cells are constructed by sandwiching two pre-cut double-sided adhesive tapes between the sample and a glass coverslip. The sample consists of EBL-patterned PEDOT:PSS, prepared from a ∼90 nm spin-coated film, on an ITO-coated glass substrate. The sample area is larger than the coverslip area. An electrolyte pipetted onto the sample near the periphery of the coverslip is drawn into the flow cell by capillary action. The sample, on the conductive ITO substrate, serves directly as the working electrode (WE). The counter electrode (CE) (platinum wire) and the reference electrode (RE) (silver/silver-chloride pellet, Ag/AgCl pellet) are in contact with the exposed electrolyte (PBS) droplet that is at the periphery of the flow cell. A potentiostat (BioLogic SP-200) is used to control the voltage that is applied relative to the reference. This system allows for electrochemical switching while maintaining optical access for microscopy.

## Supplementary Material

Supplementary Material Details
