# Supplementary material for: Direct electron beam patterning of electro-optically active PEDOT:PSS
Source: Nanophotonics. 2024 Jan 4;13(12):2271–80. doi: 10.1515/nanoph-2023-0640 (PMC11104293; doi:10.1515/nanoph-2023-0640)
Supplement: Supplementary file 1 — Supplementary Material Details [file j_nanoph-2023-0640_suppl_001.pdf]

Supplementary Information:

Direct electron beam patterning of electro-optically active PEDOT:PSS

Siddharth Doshi[1,3], Dominik Ludescher[2]*, Julian Karst[2], Moritz Flöß[2], Johan Carlström[3], Bohan Li[3], Nofar Mintz Hemed[1], Yi-Shiou Duh[3], Nicholas A. Melosh[1], Mario Hentschel[2], Mark Brongersma[3], Harald Giessen[2]*

[1]Department of Materials Science and Engineering, Stanford University, Stanford, California 94305, United States

[2]4th Physics Institute and Research Center SCoPE, University of Stuttgart, Pfaffenwaldring 57, 70569 Stuttgart, Germany

[3]Geballe Laboratory for Advanced Materials, Stanford University, 476 Lomita Mall, Stanford, CA 94305, USA

* These authors contributed equally to this work

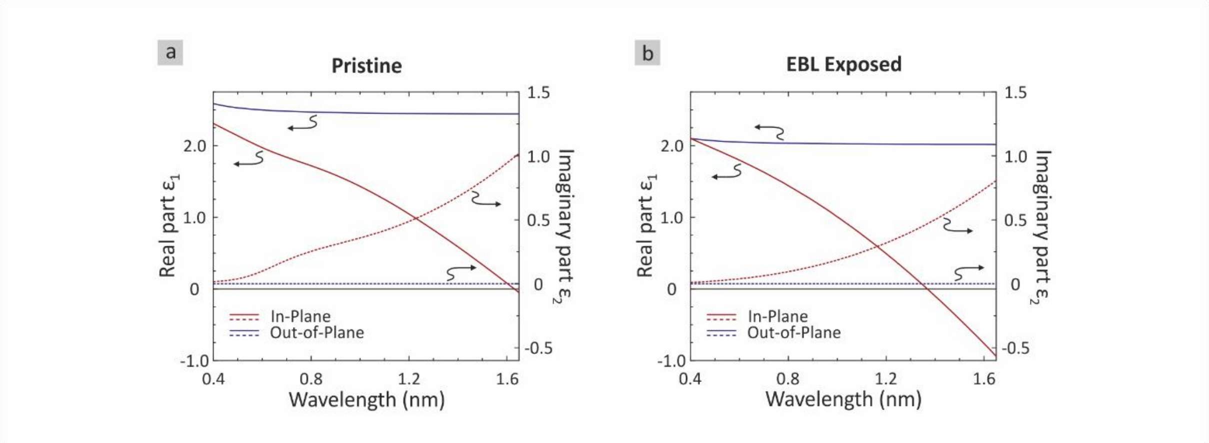

Figure S1: Optical constants ε_1 and ε_2 as a function of wavelength in the pristine and EBL exposed state. Presentation of the real part (solid line) and imaginary part (dotted line) of the dielectric function $\varepsilon = \varepsilon_1 + i\,\varepsilon_2$ for the pristine state (a) and the electron beam exposed material (b). The optical properties are obtained from variable angle spectroscopic ellipsometry fitted with an anisotropic generalized oscillator approach. Consequently, the results for the in-plane material axis (red) and the out-of-plane material axis (blue) are plotted. The measurements were modelled between 400 nm and 1650 nm.

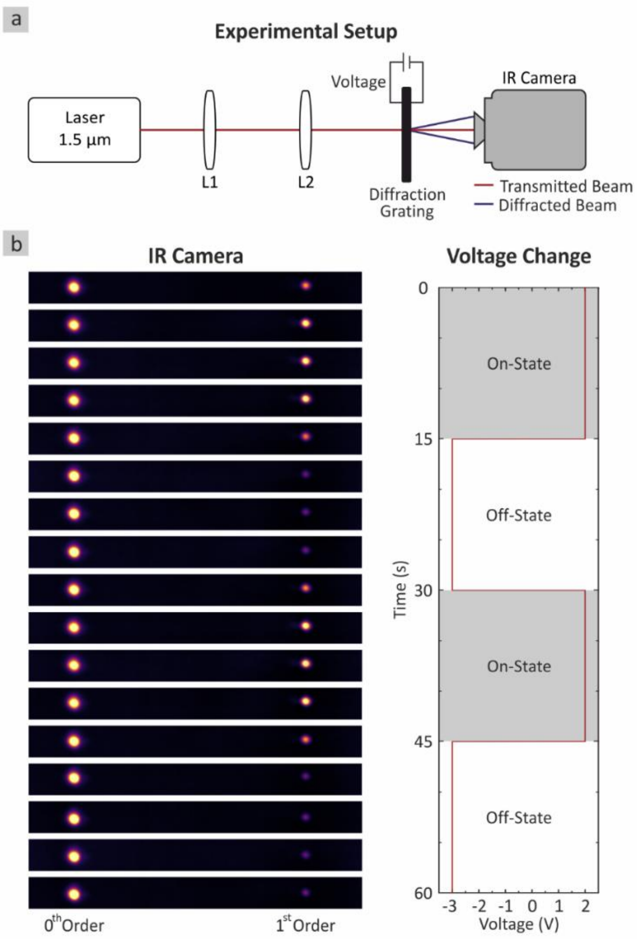

Figure S2: Experimental setup and electrical switching between the metallic and dielectric state. (a) Sketch of the experimental setup for imaging of the diffraction pattern. The laser is set to a wavelength of 1.5 µm and a telescope consisting of two lenses L1 and L2 is used to adjust the beam diameter to fully illuminate the diffraction grating which is placed into the beam path. To adjust the applied voltage, an electrochemical cell is used giving the possibility to turn the diffraction grating on and off. An IR camera is utilized to capture the transmitted beam (red) and diffracted beam (blue). (b) Chosen subsequent IR camera images showing two full switching cycles when the applied voltage is changed between +2 V (metallic) and -3 V (dielectric). By changing the applied voltage, the intensity of the diffracted beam can be varied.

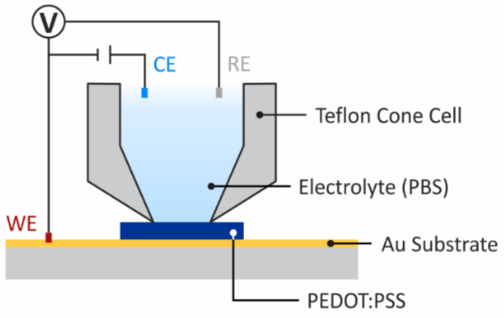

Figure S3: Experimental setup for electrochemical characterisation. Electrochemical potentials were applied using a three electrode set-up in a phosphate buffered saline (PBS) electrolyte using a Teflon capture cell. PEDOT:PSS was uniformly spin coated, or EBL-patterned on an Au coated (100 nm thickness) Si substrate which served as a working electrode (WE). A platinum wire and an Ag/AgCl pellet electrode served as the counter electrode (CE) and reference electrode (RE) respectively. Chemically cross-linked PEDOT:PSS samples covered the entire substrate, whereas the EBL-patterned PEDOT:PSS had dimensions of 11 mm x 11 mm. The Teflon capture cell allowed the measurement area to remain consistent between both samples by limiting electrolyte exposure to the interior of the cone cell (9.6 mm diameter).

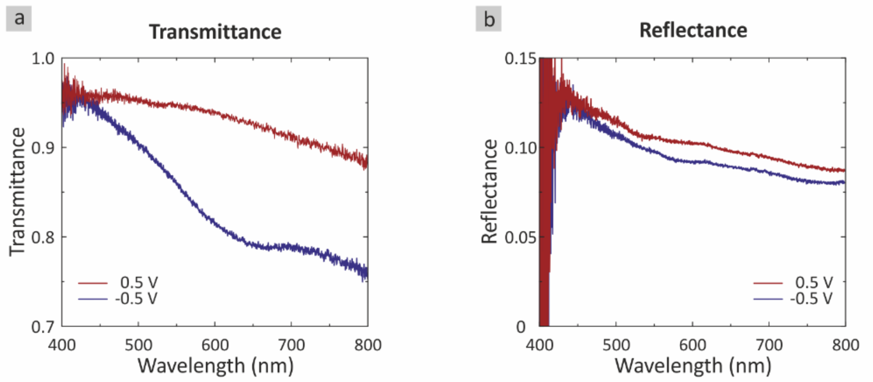

Figure S4: Gated transmittance and reflectance spectra for EBL-exposed PEDOT:PSS. (a) Transmittance and (b) reflectance spectra of EBL-patterned structures were measured using a confocal microscope (Nikon C2) coupled to a spectrometer (Acton 2300i, SpectraPro) with a cooled CCD (Princeton Instruments, Pixis 1024). A halogen lamp was used for broadband white light illumination. The structures were viewed through a coverslip corrected objective (Nikon NA 0.4, 20x) and gated electrochemically using a three-electrode set-up in a flow-cell, as illustrated in Fig 1B. The spectra are normalized by subtracting the CCD dark counts from the experimental spectrum, then the difference is divided by the spectrum of the halogen lamp collected for (a) via a bare ITO sample covered by electrolyte and a coverslip and (b) via an Ag (silver) mirror used as a reflective reference standard (Thorlabs).

Supplementary Text S1:

In these measurements, the PEDOT:PSS films had thicknesses of circa 90 nm. However, thicker films can be achieved by increasing the concentration of dissolved PEDOT:PSS or by decreasing the rotation speed utilized for the coating process. We have been able to generate conducting polymer films with thicknesses of up to 450 nm that can be patterned by increasing the applied dose. In the case of a film with a thickness of 450 nm, the deposited dose in the conducting polymer has to be doubled compared to the 90 nm thick film to develop sufficiently well-defined structures.
